# Supplementary material for: Invasiveness of Escherichia coli Is Associated with an IncFII Plasmid
Source: Pathogens. 2021 Dec 20;10(12):1645. doi: 10.3390/pathogens10121645 (PMC8707275; doi:10.3390/pathogens10121645)
Supplement: Supplementary file 1 [file pathogens-10-01645-s001.zip › pathogens-1500417-supplementary.pdf]

Supplementary Materials: The following are available online at www.mdpi.com/xxx/s1, Table S1: Patient characteristics; Figure S1: Comparison virulome of the *E. coli* strains from P1 (Eco_b1, Eco_r1), J53 and J53 after plasmid transfer (J53pEco_b1); Figure S2: Content of the IncFII_1 plasmid reconstructed by comparison of the genetic content of Eco_b1 and Eco_b1cured.

	age (years)	gender
P1	60	female
P2	28	female
P3	71	female
P4	71	male
P5	64	male

Table S1. Patient characteristics.

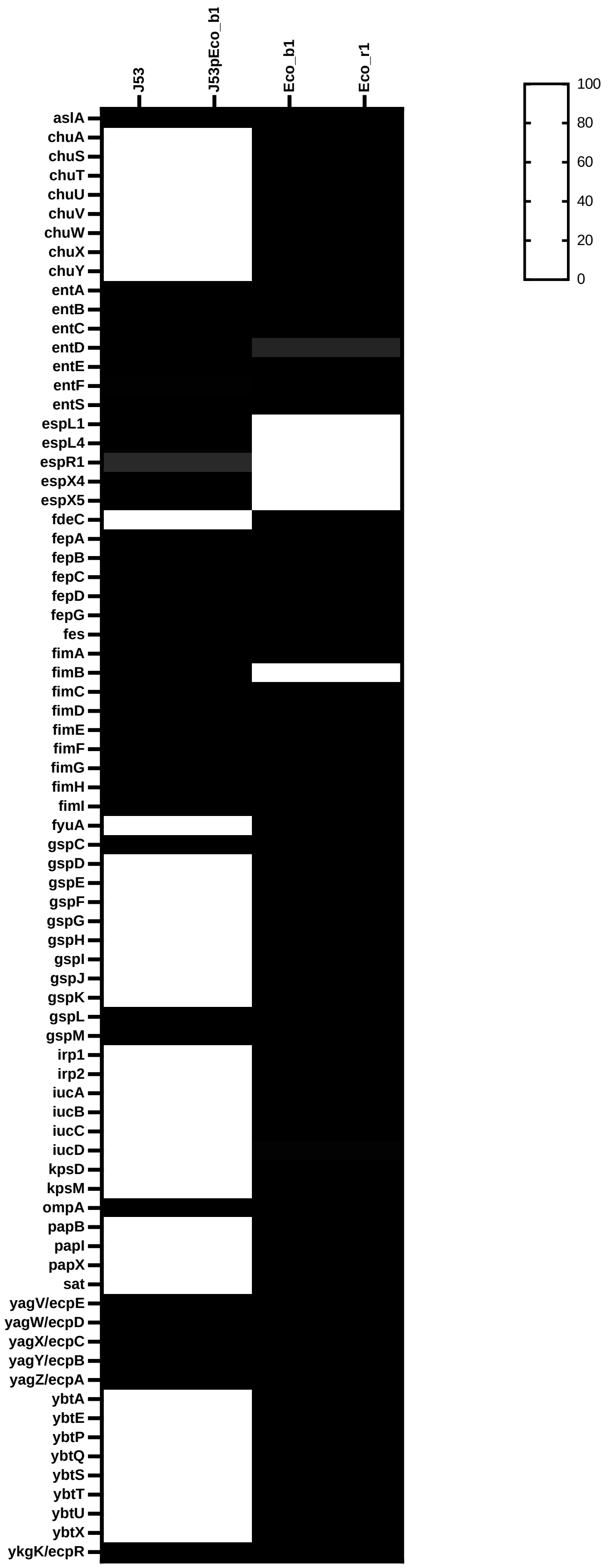

Figure S1. Comparison virulome of the E. coli strains from P1 (Eco_b1, Eco_r1), J53 and J53 after plasmid transfer (J53pEco_b1).

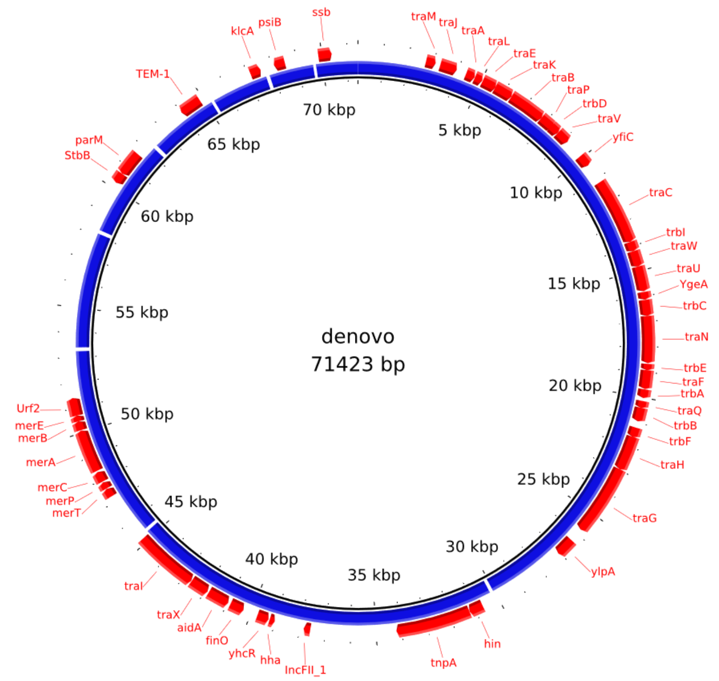

Figure S2. Content of the IncFII_1 plasmid reconstructed by comparison of the genetic content of Eco_b1 and Eco_b1cured. Each contigs absent from Eco_ b1cured were concatenated to evaluate the plasmid content.
